# A retrospective study on the aetiology, management, and outcome of brain abscess in an 11-year, single-centre study from China

**DOI:** 10.1186/1471-2334-14-311

**Published:** 2014-06-06

**Authors:** Chenran Zhang, Liuhua Hu, Xiaojun Wu, Guohan Hu, Xuehua Ding, Yicheng Lu

**Affiliations:** 1 Department of Neurosurgery, Changzheng Hospital, Second Military Medical University, No. 415, Feng-Yang Road, Shanghai 200003, China; 2 Department of Cardiology, Renji Hospital, Shanghai Jiaotong University School of Medicine, Shanghai, People’s Republic of China

**Keywords:** Brain abscess, Chronic otitis media, Streptococcus milleri, Prognosis

## Abstract

**Background:**

Brain abscesses continue to pose diagnostic and therapeutic challenges in developed and developing countries. Their aetiology and management remain complex and unclear, making improvement of treatments and outcome difficult.

**Methods:**

To determine the demographics, management, and the variables that affect the outcome in subjects with brain abscesses treated at a single centre over an 11-year period, we retrospectively analysed data in 60 patients with brain abscesses surgically treated with stereotactically guided aspiration or open craniotomy excision in Shanghai Changzheng Hospital between January 2001 and December 2011. Such variables as age, gender, Glasgow Coma Scale (GCS) score at admission, clinical presentation, location, number of lesions, predisposing factors, mechanism of infection, aetiological agent, and therapy were analysed independently.

**Results:**

Our analysis demonstrated that patient age and gender were factors that influence the occurrence of brain abscess; female patients and patients greater than 40 years of age were most likely to suffer a brain abscess. We also found that a patient’s GCS score upon admission did not influence outcome. While frequency of successful culturing of the infectious agent was low, positive cultures were obtained in only 8 of the cases (13.33%), in which the most common isolate was Streptococcus milleri. Outcome was favourable in 78.33% of the subjects, while the mortality rate was 20%. The outcome of one patient was poor due to the abscess in the basal ganglia region.

**Conclusions:**

Stereotactically guided aspiration is an effective treatment for brain abscess with an overall favourable outcome. Mortality due to brain abscess was not directly related to surgery nor surgical technique. Additional studies will continue to reveal patients trends that may improve treatment for brain abscess.

## Background

Brain abscesses occur often in the developed world, with an incidence of up to 2% of all space occupying lesions. They are even more common in developing countries, with an incidence of up to 8% [1].

Two primary treatments are used to manage brain abscesses: stereotactically guided aspiration and open craniotomy excision. Stereotactically guided aspiration is the therapy of choice over open craniotomy excision because it is less invasive, thus reducing the likelihood of neurological sequelae. Despite the advent of modern neurosurgical techniques, including stereotactic brain biopsy and aspiration, better culturing techniques to identify the infectious agent, new antibiotics, and modern non-invasive neuroimaging procedures, brain abscess still poses a public health challenge, especially in developing countries. The microbiological spectrum has changed, with increasing numbers of immune-compromised patients developing such abscesses [2,3]. On the whole, the diagnostic and therapeutic challenges posed by the intracranial abscesses are enormous.

This report is based on our experience with 82 brain abscesses surgically managed in 60 patients over an 11-year period at one single hospital. We analysed the prognostic factors and strategies of treatment to look for trends in occurrence, prognosis, predisposing risk factors, and infectious agent in brain abscesses.

## Methods

### Patient cohort

Sixty patients with a total of 82 brain abscesses verified by postoperative pathology were treated surgically between January 2001 and December 2011 at the department of Neurosurgery in Shanghai Changzheng Hospital. Demographic data, neurological status at admission, clinical presentation, predisposing factors, anatomical location, number of lesions, surgical techniques, organisms cultured, and the neurological outcome were collected from the electronic hospital data (Table 1). Written approval for this study was obtained from the ethics committee of Shanghai Second Military Medical University. All patients or their family members signed a written consent in accordance with the ethical committee standards during their hospital stay or outpatient follow-up. For patients that died, consent was signed by a family member at the conclusion of their hospital stay. Patients were excluded from the study if, 1) there was evidence of neurological symptoms unrelated to brain abscess, 2) there was evidence showing the patient had not undergone a drainage procedure or intraoperative pus sampling, or 3) the patient was lost to follow-up during the study period.

**Table 1:** Predictors of unfavorable outcomes in intracranial abscesses

**Variables**	**Favorable outcomes**	**Unfavorable outcomes**	**Total**	***p *** **Value**	***P, Regression***
	**(n = 47)**	**(n = 13)**	**(n = 60)**		**(OR, 95% CI** ***)***
**Age, y, Mean ± SD**	46.06 ± 16.97	51.92 ± 16.47		0.272	
**Sex, n (%)**				0.015	0.006 (14.003, 2.129-92.081)
Male	40 (85)	7 (15)	47		
Female	7 (54)	6 (46)	13		
**GCS on admission, no. (%)**				0.480	
≤13	5 (63)	3 (37)	8		
14-15	42 (81)	10 (19)	52		
**Clinical menifestation, no. (%)**				0.802	
Nausea	10 (71)	4 (29)	14		
Headache	35 (76)	11 (24)	46		
Fever	24 (77)	7 (23)	31		
Focal neurological sign	11 (65)	6 (35)	17		
Epilepsy	14 (82)	3 (18)	17		
**Predisposing risk factor, no. (%)**				0.050	
Postneurosurgery	6 (60)	4 (40)	10		
Post head trauma	6 (100)	0 (0)	6		
Congenital heart disease	5 (100)	0 (0)	5		
Immunosuppression	0 (0)	2 (100)	2		
COM	7 (88)	1 (12)	8		
Unknown	23 (79)	6 (21)	29		
**No. of abscess, no. (%)**				0.028	
Single	39 (85)	7 (15)	46		
Multiple	8 (57)	6 (43)	14		
**Location of abscess, no. (%)**				0.555	
Basal ganglia	4 (67)	2 (33)	6		
Frontal	14 (74)	5 (26)	19		
Temporal	12 (92)	1 (8)	13		
Occipital	7 (70)	3 (30)	10		
Cerebellar	2 (67)	1 (33)	3		
Parietal	8 (89)	1 (11)	9		
**Mode of operation, no. (%)**				1.000	
STA	27 (75)	9 (25)	36		
OCE	14 (78)	4 (22)	18		
Mastoidectomy + OCE	5 (83)	1 (17)	6		

OR, odds ratio; CI, confidence interval. The cutoff in the univariate analysis was P < 0.20. A p < 0.05 was considered statistically significant.

### Magnetic resonance imaging (MRI)

**Figure 1:**
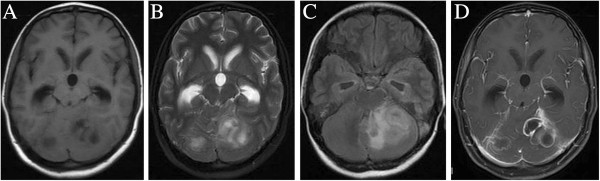
MRI showing Axial T1 hypointense (A), T2 hyperintense (B) signal, DWI (C) and Gadolinium contrast-enhanced (D) image of multiple cerebellar abscesses.

Preoperative computed tomography (CT) and magnetic resonance imaging (MRI) scans with enhancement were obtained for all patients. For conventional MRI, pyogenic brain abscesses were identified by hypointense signal in T1WI and hyperintense signal in T2WI, with ring-shaped enhancement and extensive surrounding oedema (Figure 1). Conventional MRI with diffusion weighted imaging (DWI) and magnetic resonance spectroscopy (MRS) were performed when it was difficult to discriminate brain abscesses from cystic or necrotic tumours. MRS spectra in patients with abscess showed lactate, amino acids (including valine, alanine, and leucine), and acetate peaks, while spectra for patients with cystic or necrotic tumours showed only lactate peaks. Hyperintensity was seen in all the pyogenic abscess cavities and hypointensity was observed in all the cystic and necrotic tumours on diffusion-weighted images.

### Patient evaluation

A predisposing factor was defined as any event or condition that was directly related to the onset of a brain abscess. Routes of transmission were classified as infection originating through haematogenous spread, contiguous spread, neurosurgical procedures, open head injury, or unknown causes. The neurological status at admission was evaluated using the Glasgow Coma Scale (GCS). The outcome of the patients was assessed using the Glasgow Outcome Scale (GOS), graded with a five-point score at 6 months after surgery at the outpatient clinic. A score between 0 to 3 points was considered a poor outcome and a score between 4 to 5 points indicated a good outcome. In all cases, standard laboratory tests were conducted, including a complete blood count, ESR, CRP, blood cultures, and serum chemistry. Case findings were based on the review of microbiology laboratory data for all intracranial samples. All intracranial pus samples were transported promptly to the microbiology laboratory at Changzheng Hospital microscopy, aerobic, anaerobic & fungal culture, and susceptibility testing by the laboratory’s standard protocol [4].

### Antibiotic treatment

Initial empirical antimicrobial therapies were selected in accordance with the portal of entry and the anatomical location of the abscess. Initial empirical antimicrobial therapy included a combination of vancomycin, ceftriaxone and metronidazole. Between 3 and 5 days later, treatment either remained the same or was changed based upon the results of susceptibility testing. Antibiotic therapy lasted for 4–8 weeks in accordance with the therapeutic response and neuroimaging findings. Infectious disease specialists regularly supervised this treatment.

Low-dose corticosteroid (intravenous Sou-Medrol, given in a dosage of 40 mg/12 hours) was used to manage perilesional oedema in cases of considerably significant mass effect. However, because this drug can lead to reduced MRI contrast enhancement, delayed capsule formation, increased risk of intraventricular rupture and decreased antibiotic penetration, administration was discontinued as soon as the patient was stabilized. Seizure prophylaxis or antiepileptic medication was given in all cases and continued for extended periods.

### Procedures

Stereotactically guided aspiration was recommended for abscesses >2.5 cm, signs of brain herniation secondary to space-occupying lesions or ventricular proximity, abscess growth during medical therapy or uncertain aetiology associated with neurological deterioration. This procedure was not recommended in the presence of cerebritis without imaging evidence of necrosis or capsule formation, or when the lesion location is inaccessible. This procedure was carried out in lesions in the vicinity of or at eloquent brain areas, in deep locations (brain stem or basal ganglia region) or ventricular proximity and in patients with multiple abscesses or with a poor general condition. If the size of the abscess on CT or MR images obtained after the first aspiration increased or was not reduced despite antibiotic therapy, aspiration was repeated. During surgical procedure, the abscess was drained completely and rinsed with saline containing gentamicin until the effluent was clear. When the abscess broke into ventricular space, leading to ventriculitis, external ventricular drainage or lumber puncture combined with intrathecal or intraventricular injection of sensitive antibiotic was performed.

Patients with poor response to repeated aspirations and medical treatment underwent complete excision of abscesses through open craniotomy excision. Open craniotomy excision was indicated for most brain abscess that were secondary to open head injury to remove bone chips, foreign bodies, and devitalized tissue, in multiloculated brain abscesses that are more likely to have recurrence, in deep-seated abscesses, and in fungal aetiology lesions. Postoperative abscesses where burr hole aspiration would hinder the fusion of the bone flap also underwent complete abscess excision through open craniotomy excision. Patients with chronic otomastoiditis and cerebellar abscess underwent radical mastoidectomy and open craniotomy excision.

## Results and discussion

Eighty-two abscesses were diagnosed and surgically managed in 60 patients. Brain abscesses were singular in 76.67% of the subjects and multiple in 23.33%, a result similar to that reported by Landriel F [5]. The mean patient age was 47.33 ± 2.18 years (range 9–76). The male-to-female ratio was 3.62:1. The most frequent clinical presentations included headache, fever, and focal neurologic deficits. Headache occurred in 76.67% of the subjects. Similar results have been reported in several other studies. Fever and focal neurologic deficits were also common, occurring in 51.67% and 28.33% of the patients, respectively. Despite being the most common, the “classic” triad of headache, fever and focal neurological deficits was seen together in only 5% (n = 3) of the cases. From the data presented here, the classic presentation described by Renton *et al.* is helpful, but actually has low sensitivity [5-12].

Fourteen patients had nausea and 17 patients presented with epilepsy. Seizures were present in the 28.33% of the cases, comparable to incidence rates presented in other large analyses [13,14]. Four patients complained of visual disturbance, which was connected with occipital lesions. Aphasia was observed in two patients. In this study, 52 of the 60 patients had good GCS scores at admission, which had no effect on the clinical outcome. The symptoms with which patients presented upon admission did not correlate with the GOS results at 6 months.

The patients within this cohort had a variety of predisposing factors and comorbidities. Eleven patients had adjacent localized cranial infection, chronic otitis media (COM), paranasal sinusitis, or tooth abscess. The mostly common predisposing factors included post-neurosurgery, COM, and congenital heart disease (in 5 patients); however, 29 patients had no identifiable predisposing risk factors. Two patients were immunosuppressed upon admission. Sixteen patients had posttraumatic or postoperative abscesses. Twenty-nine patients had no identifiable predisposing cause.

The frontal lobe was the most common abscess location in the patients, followed by the temporal and occipital regions. However, in a study carried out by Cavuşoglu H *et al.*, the temporoparietal region was the most commonly affected location [14]. Abscesses of unknown cause accounted for 48.33% of the subjects, a percent higher than the values reported for other series [5-7,10,15].

The route of transmission was contiguous infection in 18.33% (n = 11) of the cases. This property directly influence which region of the brain was affected (Table 2). For example, otogenic infections were associated with temporal or cerebellar abscesses. However, 41.18% of the frontal lesions had unknown predisposing causes. In most large series of brain abscesses from developing countries, middle ear infection has been reported to be the most common source of intracranial suppuration, a result similar to that found here. However, there has been a decline in developed countries in cases of COM [16].

**Table 2:** The site of the abscess in relation to the underlying cause

**Predisposing factor (N)**	**Location of abscess**	**No. of patients (n ≥ 2)**
COM (8)	Temporal	6/8
COM (8)	cerebellar	2/8
Immunosuppression (2)	Frontal	2/2
Unknown (29)	Frontal	7/29
Unknown (29)	Occipital	8/29
Postneurosurgery (10)	Frontal	4/10
Postneurosurgery (10)	Basal ganglia	3/10
Post head trauma (6)	Frontal	3/6

In 60% of the cases, the treatment of choice was stereotactically guided aspiration, while 30% of the cases were managed through open craniotomy excision. Six patients were treated by performing radical mastoidectomy combined with open craniotomy excision therapy. One patient was immunosuppressed due to liver transplantation. His left frontal abscess eroded into the skull base and ethmoid sinus. He was treated by performing an intranasal ethmoidectomy and further aspiration.

The results of these surgical techniques presented no significant differences in terms of outcome or associated complications. However, the effectiveness of stereotactically guided aspiration and open craniotomy excision differs across multiple reports. Xiao *et al.* reported similar effectiveness between the two procedures, but significantly lower mortality (p = 0.02) with open craniotomy excision [7]. Mampalam *et al.* reported less frequent brain abscess recurrence in open craniotomy compared with stereotactic aspiration [17]. Ratnaike TE *et al.* reviewed the Ovid Medline database in a range from 1990–2009 to identify all articles relating to brain abscess. They found the high mortality rates from aspiration in the pre-computer tomography era decreased dramatically after computer tomographic scanning became available. In their review, the mean mortality for aspiration post-1990 was 6.6% for publications with more than five patients. With surgical excision by craniotomy, the mean mortality in the same period was 12.7%. They concluded that aspiration may be the first surgical choice in patients with supratentorial parenchymal brain abscesses [18].

A multivariate analysis was performed to determine which patient factors were associated with poor outcome (Table 1). The tested variables included gender, age, GCS on admission, symptoms, pre-disposing risk factors, location of abscess, and mode of operation. In this analysis, 86.67% of the patients had a good GCS score at admission, which had no bearing on the clinical outcome. One reason why GCS score was unrelated to outcome may be because some patients with high GCS scores were immunocompromised or had accompanying primary lethal disease, such as glioblastoma. Similarly, a study by Landriel F, 79.6% of the patients had a good GCS score at admission [5].

In light of these results, no conclusions can be drawn regarding the effect of neurological deterioration on the outcome; however, there is adequate evidence that this is one of the most important factors determining prognosis [6,7,13]. Previous studies have found an altered level of consciousness present in up to two-thirds of patients [17,19]. In Manzar N’s study, 69.8% of the patients were brought to the hospital with an altered state of consciousness [15]. The level of consciousness at presentation has been shown by other authors to be of great prognostic value [14,20].

The male-to-female ratio in our study was 3.6:1; that is, males were found to be more affected than females in the present study, irrespective of the age group. Similar observations have been reported in different parts of the world [15,21,22]. However, Landriel F reported 59.3% of the patients with intracranial abscesses were female [5]. Despite a greater occurrence in males in this cohort, the analysis revealed that the female gender was associated with poor outcome. Because of the small sample size, these findings should be verified in a multicentre trial in the region and a larger sample size.

Additionally, this analysis revealed that patients older than 40 years were most susceptible to brain abscess. Overall, 42 (70%) subjects were older than 40 years. These results are similar to a previous report showing that patients older than 40 years are most susceptible to brain abscess [5]. On the contrary, some authors reported that brain abscess occurs more frequently in the younger age group, usually during the first three decades of life [5,23-25]. In Manzar N’s study, 34 (64.2%) subjects were older than 15 years and the majority of patients were younger than age 40 years [15]. Similarly, in a study carried out by Sinha *et al.*, 74.89% of the patients were younger than 40 years [22]. Overall, it is clear that the age group most affected by brain abscess is not yet clear from the available data. There may be additional, underlying factors involved in predisposition to brain abscess at any age.

Laboratory data had little diagnostic or prognostic value (Table 3). Twenty-five patients had elevated peripheral white blood cell counts, all with a predominant leucocytosis. The erythrocyte sedimentation rate was found to be increased in 12 patients. However, C-reactive protein levels were found to be normal in 51.7% of the patients.

**Table 3:** Major demographic and laboratory findings of patients

**Characteristic of patients**	**No. of patients (n = 60)**	**Frequency (%)**
Age (years)		
≤40	20	33.3
>40	40	66.7
Sex		
Male	47	78.3
Female	13	21.7
GCS on admission		
≤13	8	13.3
14-15	52	86.7
Raised lab parameters		
ESR	12	20
CRP	29	48.3
WBC	25	41.7
No. of abscess		
Single	46	76.7
Multiple	14	23.3

Because many patients were receiving antibiotic therapy prior to surgery, positive cultures were obtained in only 8 of the cases, in which the most common isolate was *Streptococcus milleri* (Table 4). In all 8 cases, a single organism was detected suggesting monomicrobial aetiology, and the same trend has been noted in various studies throughout the world [15,26-28]. Negative cultures totalled 86.67%, which is much higher than the rates observed by other authors [5,13-15,29]. Even when culturing results are negative, empiric therapy must be administered against the suspected causative agent. There are several possible reasons for the high culture negative rate. First, antibiotic management policies in China are relatively loose, and the preventative use of antibiotics is very common. Second, the intracranial pus samples may not have been transported to the microbiology laboratory quickly enough to successfully analyse. Finally, the standard culturing protocols used in this hospital could have missed certain organisms, such as anaerobic bacteria.

**Table 4:** Culture-positive bacterial isolates from patients with brain abscesses

**Organism**	**No. of patients (n = 60)**
Streptococcus intermedius	1
Ps. aeruginosa	1
Streptococcus sanguis	1
Streptococcus oralis	1
G + coccus	1
Streptococcus alactolyticus	1
Streptococcus anginosus	1
Streptococcus pneumoniae	1
No growth	52

Saito *et al*. diagnosed culture-negative brain abscesses with *Streptococcus intermedius* through direct nucleotide sequence analysis of the 16 s ribosomal RNA gene [30]. Al Masalma *et al.* performed a 16S rDNA-based metagenomic analysis of cerebral abscesses from patients diagnosed from 2006 through 2010. By detecting polymicrobial infections in 19 patients, their strategy was significantly more discriminatory and enabled the identification of a greater number of bacterial taxa than did culture and conventional 16S rDNA polymerase chain reaction (PCR) and sequencing, respectively (P < 0.01). They concluded that cloning and sequencing of PCR-amplified 16S rDNA is a highly valuable method to identify the bacterial species found in brain abscesses [31]. Bajpai A [32] compared the conventional culture, PMRS and molecular method (16S rRNA PCR and sequencing) for the detection of aerobic and anaerobic bacteria in brain abscess. They found that, being a non-invasive modality, *in vivo* PMRS is useful for the rapid categorization of bacteria with reasonable specificity and sensitivity. It can categorize bacteria into anaerobes or aerobes even in sterile samples as well as for the bacteria which are relatively slow growing or difficult to identify by conventional methods.

Of the 60 patients included in this study, outcome was favourable in 78.33% of the subjects. The outcome of one patient was unfavourable because abscess formed after evacuation of hematoma in the right basal ganglia region. The mortality rate in our study was 20% (12 cases). Ten patients died in the immediate postoperative period from complications and 2 patients who had been operated for a glioblastoma died after discharge. Of the 10 patients who died in hospital, two were immunosuppressed at the time of diagnosis and died from multisystem organ failure. Two mortalities were the result of lethal complications not directly related to the surgical procedure: hematoma in the midbrain and operative area, respectively. In three patients, the abscesses broke into the ventricular system and the patients died from ventriculitis and infectious shock. The remaining three deaths were due to postoperative brain herniation from high intracranial hypertension.

The mortality rate shown here is similar to the rates observed by other authors, which range between 8% and 53% [11]. Manzar *et al.* reported that the most important factors influencing mortality was the neurologic condition of the patient at the time of admission [15]. Landriel *et al.* revealed that age, immunosuppression and haematogenous spread were all associated with poor outcomes [5]. In contrast, our study revealed that only gender was a critical factor influencing outcome. As far as therapeutic effect is concerned, Park SH *et al.* demonstrated that MRI plus FDG-PET improved the accuracy of assessing therapeutic responses to antibiotics treatment of brain abscess and aided in optimizing therapy [33].

Seizures were the most common form of post-operation complication and were treated with thorough anti-epileptic therapy. After discharge from the hospital, the patients were monitored at the outpatient clinic for 7 ~ 19 months. After 15 months, 12 patients were lost to follow-up and of the remaining patients who could be assessed, neurological scores remained unchanged. At the end of the follow-up period, the surviving patients showed no clinical or neuroimaging signs of recurrence.

## Conclusions

The retrospective study present here revealed a number of interesting trends in the aetiology, treatment options, and patient populations of brain abscesses. Patients older than 40 years were the most susceptible to brain abscess. Male gender was the only predictor of favourable prognosis. Mortality due to brain abscess was not directly related to surgery or surgical technique. Early diagnosis and appropriate management will lead to a better prognosis. However, a large-scale prospective multi-centre study is recommended for further evaluating the ethnic and geographic differences and other risk factors for brain abscesses in underdeveloped countries like China.

## Competing interests

The authors declare that they do not have any financial or non-financial competing interests.

## Authors’ contributions

ZCR and LYC conceived the concept and design of the study. Data was gathered by HLH and WXJ. ZCR and DXH performed the data analysis. ZCR, HLH and HGH wrote the manuscript. All authors read and approved the final manuscript.

## Pre-publication history

The pre-publication history for this paper can be accessed here:

http://www.biomedcentral.com/1471-2334/14/311/prepub
